# Insights into Canadians’ Perceptions of Service Dogs in Public Spaces

**DOI:** 10.3390/ani13193091

**Published:** 2023-10-03

**Authors:** Maryellen Gibson, Linzi Williamson, Colleen Anne Dell

**Affiliations:** 1 Department of Sociology, University of Saskatchewan, Saskatoon, SK S7N 5A2, Canada; colleen.dell@usask.ca; 2 Department of Psychology and Health Studies, University of Saskatchewan, Saskatoon, SK S7N 5A2, Canada; linzi.williamson@usask.ca

**Keywords:** Service Dogs, public perceptions, Veterans’ wellness, assistance animals

## Abstract

**Simple Summary:**

Veterans living with posttraumatic stress injuries (PTSIs) are increasingly being paired with service dogs (SDs) to manage their symptoms. SDs have public access in Canada and are found in nearly all types of settings, ranging from a restaurant to a dental office. Negative perceptions of SDs amongst the public are a concern for Veterans, as they may contribute to negative interactions with them. This study sought to understand the general public’s perceptions of SDs in Canada through an online questionnaire. Four hundred eighty-five people shared their personal characteristics and what they felt about SDs in public spaces. We found that the Canadian public holds highly positive perceptions of SDs in public spaces, with some groups (such as women) being more supportive than other groups (including certain cultural backgrounds). This is important information to have gathered for the future development of targeted public awareness campaigns.

**Abstract:**

Service Dogs (SDs) are an increasingly common type of working dog supporting people with disabilities in Canada. One of the groups being paired with SDs is Veterans diagnosed with posttraumatic stress injuries (PTSIs). In past research, Veterans have expressed stress over negative interactions with people in public spaces because an SD brings attention to their disability. There is a dearth of research exploring perceptions of SDs in public settings. Methods: A total of 485 Canadians were surveyed via an online questionnaire about their demographic information and beliefs about SDs in public spaces. Data were analyzed using robust ordinary least squares (OLS) regression to determine which demographic features, if any, contribute to perceptions. Results: Generally, the Canadian public holds highly positive perceptions of SDs being in public spaces. Our analysis found that women, people who currently have pets, and Indigenous people were more supportive of SDs in public spaces than others. People with certain cultural heritages were less receptive. Conclusion: These findings are an important beginning contribution to the growing SD and Veteran health field.

## 1. Introduction

Service Dogs (SDs) are one type of working dog that is increasingly present in our communities. There is concern among animal welfare and disability advocates around the variety of working dogs (i.e., therapy dogs, emotional support dogs, and Service Dogs) that are increasingly present in public and private spaces and the potential confusion or lack of support from the general public regarding Service Dogs in public spaces [1]. SDs are distinguished from other working dogs by performing at least one identifiable task that helps to support an individual with a disability (e.g., glucose level detection, waking from nightmares) and should be trained to a higher standard [2]. SDs are trained to perform a variety of disability-support tasks for people with physical, sensory, neurological, and developmental/cognitive impairment or other daily challenges [3]. Generally speaking, people have the legal right to access public spaces accompanied by their Service Dog anywhere the public is allowed as long as the presence of a dog does not significantly compromise the health and safety of others; this has been termed Service Dog public access and differs slightly among provinces and territories in Canada [4,5]. This is different from other types of working dogs, such as emotional support animals (ESAs), those providing emotional benefit to their handler without specific training, and therapy dogs, which are those who visit with individuals outside their home to provide comfort [2]. ESAs are legally supported through “reasonable accommodation” to be with their handler in public spaces, but that handler often requires a medical note of support (Howell and Bennett, 2022, pp. 7). Therapy dogs, however, are only allowed in public spaces where they have been invited to visit. It should be noted that these working dogs also differ from companion animals, otherwise known as pets, who are not specifically trained nor have the right to access public spaces unless specified (e.g., restaurant patios) [2].

Given the increase in SDs in public spaces over the last five or so years, potential confusion exists among different types of working dogs. One reason for this is that the media routinely uses the terms interchangeably (e.g., therapy dog and Service Dog) and tends to report on extreme or negative confrontations between the public and working animals. Consequently, public perception studies have become more prevalent in recent years in the American and Australian settings [1,6,7,8]. There is a dearth of such studies conducted in Canada. Within the international research in this area, it has been found that, overall, perceptions of SDs are positive. Members of the general public have expressed positive feelings towards SDs, such as love, happiness, admiration, and interest [7]. Research participants have recognized that SDs provide practical benefits to their handlers, along with emotional support, and that the dogs are very skilled at assisting their handlers and seem to enjoy the work [7]. However, there is some confusion among the general public around public access legislation and the difference between Service Dogs and other forms of working dogs [1,6,9].

Despite the mostly positive reflections on SDs, research has also found that individuals with SDs can experience discrimination based on their disability given that many otherwise “invisible” illnesses (e.g., mental health concerns) become visible through the presence of an SD [8,10,11,12]. Up to 50% of individuals with an SD report experiencing some form of discrimination when in public, but those with invisible disabilities report experiencing even higher rates of discriminatory experiences [10,12]. The most common forms of discrimination reported by handlers of SDs were invasive questioning from others about their dog and their disability, unwanted attention in a public space, and questioned legitimacy of their SD’s training and the reality of their disability [12]. There is also concern with how media portrayals of negative news stories about “fake” Service Dogs, those who are not properly certified or do not have any specific training [13], may make members of the public skeptical about SDs in public spaces and lead to negative stereotyping or interactions [14]. These findings suggest that some individuals may hold negative perceptions of SDs and/or invisible disabilities or are unaware of the rights of people paired with an SD. Holding these negative perceptions or being skeptical of a service animal may lead individuals to confront or provide unwanted attention to SDs and handlers that they encounter in their daily lives. These confrontations may compound mental health symptoms. For example, individuals with a posttraumatic stress injury (PTSI; Carleton et al., 2022) such as posttraumatic stress disorder (PTSD) may experience increased hypervigilance and irritability if confronted in public while working with their SD.

PTSD is a “psychological response to the experience of intense traumatic events, particularly ones that are life-threatening” with four symptom categories: (1) intrusive thoughts (e.g., flashbacks, nightmares, etc.), (2) avoiding reminders/numbing (e.g., avoiding events or places), (3) negative thoughts and feelings (e.g., pessimism, detachment, flat affect, etc.), and (4) arousal/reactivity (e.g., hypervigilance, sleep problems, etc.) [15,16,17,18]. PTSD is often associated with other health concerns, including co-occurring substance use concerns and other mental and physical health ailments, such as depression, anxiety, and suicidality [16]. By having a visible marker of an invisible disability, individuals with conditions such as PTSD who are working with SDs may be putting themselves at risk in the current environment to be confronted with unwanted questions and attention in public spaces. There is potential that these negative interactions may increase PTSD symptoms or reduce the willingness of handlers to venture into these public spaces, further isolating themselves. Canadian Veterans are increasingly working with SDs as a complement to traditional treatment protocols for PTSD and associated health concerns [19,20], so this area requires increased attention.

Understanding which factors are likely to lead to an individual discriminating against SDs and their handlers is essential for developing targeted public education interventions. Past research has noted that females and those of certain cultural backgrounds may experience higher rates of dog phobias, suggesting that they may show lower support for public access of Service Dogs [21]. Women, however, have been found to be more knowledgeable about and trusting of SDs in public and less likely to assume that handlers with SDs are fraudulent or that the handlers are faking their illness; this finding has not been explored by others in the literature [1,2,22]. Alternatively, given that the core experience and benefits of the human–animal bond are common across SDs and companion animals [23,24], it can be hypothesized that those with past or current pet ownership may hold more favorable perceptions of SD public access.

Canadian research on public perceptions of SDs and the relationship between demographic characteristics and those perceptions is scant, and so the current project seeks to fill these gaps. Because of the reported discrimination experienced by SD handlers in public spaces, we specifically focused on how the Canadian public views the public access of SDs. This project seeks to answer the following research questions: 1) What are the public perceptions of Service Dogs in Canada? And 2) Do personal characteristics impact perceptions of Service Dogs’ public access in Canada? If yes, how? To answer our research questions, analyses were conducted on previously collected data surrounding stereotyping and perceptions of Service Dogs paired with a Veteran or first responder (see Williamson et al., 2023) [25].

## 2. Materials and Methods

### 2.1. Procedure

This study was approved by the University of Saskatchewan Research Ethics Board (BEH-2898). Data were collected via an online questionnaire that measured public stereotyping and perceptions of Canadian Veterans and first responders with varying disabilities who were paired with a SD. Data collection occurred in 2022 via Ekos/Probit (a third-party research vendor), who distributed the questionnaire to a random, stratified sample of English- and French-speaking Canadians aged 18 years and older.

The current project examined the demographic data and participant responses to questions related to SD public access, which focused on their level of agreement/support for SDs being present in a variety of settings: airports, restaurants, public transportation systems, workplaces, and medical settings. All settings were reflective of locations where SDs are legally allowed to enter under provincial/territorial Human Rights Codes across Canada [5]. These questions were answered on a five-point Likert scale (1 = strongly disagree, 5 = strongly agree). Table 1 summarizes the responses to these questions, which were combined into a composite variable (α = 0.9325) to represent the overall perception of SD access. The respondents were highly in agreement with the statements overall. This variable then became the dependent variable for the analyses. The highest levels of agreement reflected SD access to public transit (4.361), SD access to restaurants (4.579), SDs accompanying their handlers to work in office buildings (4.523), and SDs accompanying their handlers to work with seniors (4.456). The lowest level of support reflected SDs accompanying their handlers to work in the food service industry (3.444). Participants were also asked to report on several demographic variables, including their age, race/ethnicity, their own experience with personal SDs, pet ownership, personal health conditions, career affiliations, and negative experiences with dogs. The respondent demographics can be found in Section 2.2 (Participants).

In the original study (see Williamson et al., 2023) [25], participants were randomly assigned to vignette conditions that each described a Veteran or first responder who required an SD to aid with their PTSD, substance use disorder (SUD), or physical mobility limitation (PML). Regression analysis revealed no significant differences among vignette conditions (Table 2), so the entire sample was combined for subsequent analyses.

### 2.2. Participants

A total of 485 participants completed the online questionnaire (see Table 3). The questionnaire was offered in both English and French given the bilingual nature of Canada, although the majority of responses were in English (84.95%). The gender distribution included 43.3% men and roughly 52% women, with 2.89% of the responses falling into the trans or non-binary categories, which had to be combined due to small cell counts. A small minority (1.86%) of the respondents noted that they currently or previously had a SD of their own. Most respondents (53.2%) fell into the category of 31–59 years of age. The vast majority also identified as White or European, with the next largest categories being Asian (3.09%) or multiracial (2.89%). A total of 81.03% noted that they did not have a military or first responder background. Given the nature of this study, respondents were also asked about their past negative experiences with dogs and current pet ownership. A total of 46.80% had not experienced any negative interactions with a dog, but 29.28% had experienced a dog bite and 12.99% reported a fear of dogs. Over half (58.35%) of the respondents also noted that they currently owned pets. 

### 2.3. Analyses

To examine whether demographic characteristics significantly impacted the level of agreement with SD public access, these characteristics were run through robust ordinary least squares (OLS) regression to determine the direction and strength of these relationships. This was done to explore the key predictors for positive perceptions of SD public access. Predictive margin testing was then conducted to provide estimated levels of support for public access based on the statistically significant findings from the regression.

## 3. Results

As shown in Table 4, OLS regression with robust variance estimates was used to test the impacts of the available demographic information on the perception of SD public access. Women were found to be more supportive (β = 0.216) than men of SD public access. Annual income was found to have little effect on the dependent variable, but those in the second highest income range ($120,000 to $139,999) were found to be significantly less supportive (β = −0.468) than those at the lowest income level. Educational attainment was found to have no impact on perceptions. Race/ethnicity was found to have significant results, however.

Indigenous individuals reported significantly higher support for public access (β = 0.381)), followed by White or European respondents, and respondents with self-reported “other” ethnicities (Black, Middle Eastern, and Latino or Hispanic) were significantly less likely to support SD public access (β = −1.030). Personal SD ownership did not have a significant impact on the dependent variable.

Having a history of negative interaction with dogs was not found to significantly impact the dependent variable for the respondents. No impact was also found for individuals who grew up with pets and those who did not. Current pet ownership, however, had a marginally positive impact (β = 0.146) on perceptions.

In order to better understand these effects, we have also provided the margin estimates of the statistically significant variables listed above (see Table 5). The margins presented showcase the predicted value of public access support on a five-point Likert scale (1 = not at all supportive to 5 = highly supportive). With other variables held constant, women were likely to fall at 4.32 on the scale of support for SD public perceptions, whereas men were at 4.10. Indigenous respondents were at 4.61 on the scale, and “Other” respondents were at 3.19. For current pet ownership, with other variables held constant, those with pets reported 4.28 on the scale, and those without a current pet reported 4.13.

## 4. Discussion

This project sought to better understand the overall public perceptions of Canadians towards SD public access. If negative perceptions of SDs exist, this raises the possibility of negative interactions in public settings and may impact the treatment of the handlers of SDs and the symptoms they may experience. Further, negative interactions in public may also place the SDs at risk of experiencing undue stress. The welfare of the SDs in these situations should always be considered. It is important for researchers, SD organizations, policy makers, and the general public to better understand the current public perceptions of SDs and recognize where tailored educational campaigns may be beneficial. The findings from this project indicate a high degree of support for public access, as identified in similar studies in the United States and Australia [1,7]. Despite the negative experiences that Veterans and others with SDs report having experienced, the results from this study suggest that the general Canadian public is highly supportive of SDs in public spaces. Those with SDs have called for increased education about public access so as to decrease their negative interactions with civilians [12,26,27]. This education may include awareness of invisible disabilities such as PTSD, the role of Service Dogs in the lives of their handlers, what public access in Canada entails, and what individuals can expect and how best to behave when they meet a SD and handler in public spaces. This study recognizes that there is still a need to explore education and awareness about public access, especially for those groups that were found to be less supportive.

Despite research findings that suggest that women may be more likely to experience dog phobias, public perception studies have found that women have a higher degree of trust and support for SDs in public [1,21], a finding mirrored in this study. Men have historically been found to have lower mental health literacy and to rate mental health concerns as less serious than their female counterparts do [28]. Further, the work of Shoenfeld-Tacher et al. (2017) found that men were more likely to be critical of SDs and not trust that individuals with SDs actually need them. Given the increase in SDs being paired with individuals with mental health concerns and other invisible illnesses, male judgments and critical approaches toward mental health conditions may impact their support for SD public access.

Race/ethnicity appears to be a significant factor influencing perceptions of SD public access. Examining all respondents, Indigenous respondents were found to be the most supportive, White or European respondents somewhat less, and individuals in the “Other” category (including Black, Middle Eastern, and Latino or Hispanic populations) were yet less supportive. Past research has found that cultural background can have an impact on perceptions of animals in general, and these impacts may span over generations [29]. Cultural background and worldviews may influence aspects such as the culturally defined roles that dogs play in human society and spaces in which dogs may or may not be acceptable [14,21,29,30]. These factors may be impactful on perceptions about SD access and require further study.

Negative experiences with dogs, including a fear of dogs, dog allergies, and a history of dog bites, did not significantly impact perceptions of public access, as may have been predicted based on the findings of past studies. The results from this study also suggest that past or current pets and, therefore, knowledge on the human–animal bond may not be as impactful on SD perceptions. These results suggest that other factors may be more impactful than past pet ownership, with current pet ownership being significant to only a small degree.

This study also found that income may impact one’s level of support for SD public access, with the individuals who were the least supportive being those who fell into the $120,000–139,999 category. At the time of writing, no comparative data on how income level may impact the perception of SDs or related topics have been published in order to better understand this outcome. Future research may also consider qualitative data collection techniques to better understand how these factors may influence perceptions.

The findings from this project also need to be contextualized within the limitations of this study. The results are based upon self-reported perceptions, which may lead to social desirability bias, wherein respondents answer how they perceive will be most socially acceptable, rather than reporting their true perceptions. This limitation was addressed as best as possible through the collection of a random sample through a third-party vendor rather than researcher-selected participant sampling. Another limitation of this study is the small cell count for certain variables, resulting in the combined “Other” categories that include individuals from varying backgrounds. Future research may want to purposefully oversample these populations in order to better understand their perceptions. Third, the responses analyzed in this project were taken at a single time interval in the year 2022. Therefore, these results do not offer insights into changes in public perceptions over time or how these perceptions may have changed in recent years due to the increased presence of SDs, public awareness campaigns, and media coverage. Lastly, this study included vignettes of Veterans and first responders; therefore, these results may not represent public perceptions of SDs with handlers with other disabilities or health conditions such as visual impairments.

## 5. Conclusions

With more Service Dogs being paired with people with disabilities, there is a need to understand public perceptions of having SDs in public spaces given their right to public access. This exploratory study suggests that certain personal attributes may impact these perceptions and populations who may benefit from targeted awareness campaigns. Overall, however, public perceptions of Service Dog public access in Canada are highly supportive.

## Figures and Tables

**Table 1:** Responses to prompts on SD public access by percentage.

Prompt	Strongly Disagree	Disagree	Neither Agree nor Disagree	Agree	Strongly Agree	Mean Value	Std. Dev.
Service Dogs should be allowed in restaurants	1.24%	1.65%	3.09%	25.77%	67.84%	4.579	0.745
Individuals who work in a factory should be able to bring their Service Dog with them	3.09%	10.31%	20.41%	26.80%	38.35%	3.879	1.13
Service Dogs should be allowed in airplane cabins with other passengers	1.44%	3.92%	9.28%	27.01%	57.11%	4.361	0.913
Service Dogs should be allowed in public transportation vehicles (e.g., bus transit, taxis, streetcars, ferries, etc.)	0.83%	0.83%	3.12%	24.32%	70.89%	4.636	0.667
Individuals who work in an office building should be able to bring their Service Dog with them	1.03%	1.65%	6.60%	25.15%	64.95%	4.523	0.779
Individuals who work in the food service industry should be able to bring their Service Dog with them	5.15%	19.38%	27.63%	20.62%	26.60%	3.444	1.219
Individuals who work in a healthcare setting should be able to bring their Service Dog with them	3.71%	12.16%	25.57%	23.51%	34.43%	3.732	1.166
Individuals who work in a school should be able to bring their Service Dog with them	1.65%	5.15%	9.28%	28.87%	54.43%	4.301	0.954
Individuals who work with children should be able to bring their Service Dog with them	1.86%	3.92%	10.93%	29.69%	52.99%	4.289	0.942
Individuals who work with senior citizens should be able to bring their Service Dog with them	0.82%	3.30%	7.01%	26.80%	61.44%	4.456	0.83

**Table 2:** Regression analysis results of the public access support level based on vignette conditions.

Vignette Condition	β	*p*-Value
First Responder with Physical Mobility Limitation (PML)	Ref.	Ref.
Veteran with PTSD	0.018 (0.091)	0.844
Veteran with Substance Use Disorder	−0.041 (0.094)	0.660
Veteran with PML	0.135 (0.098)	0.168

Notes: *p* < 0.05; *p* < 0.01; standard deviation in parenthesis; public access as a continuous variable between 1 and 5.

**Table 3:** Personal attributes of the respondents.

Attribute		Freq.	Perc.	Attribute		Freq.	Perc.
Language	English	412	84.95	Race/Ethnicity	Indigenous to North America/Turtle Island	12	2.47
Gender	Man	210	43.30		White or European	405	83.51
	Woman	252	51.96		Asian	15	3.09
	Other Identity	14	2.89		Other	10	2.06
					Multiracial	14	2.89
	Missing	9	1.86		Missing	29	5.98
Personal Service Dog	Yes	9	1.86	Age	19–30	54	11.13
	No	475	97.94		31–59	258	53.20
	Missing	1	0.21	Career Affiliation	Armed Forces	4	0.82
Personal Health Condition	PTSD	41	8.45		Veteran	26	5.36
	SUD	14	2.89		First Responder	12	2.47
	PML	77	15.88		Other	12	2.47
	TBI	19	3.92		None of these	393	81.03
	None Listed	334	68.87	Negative Dog Experiences	Dog Allergies	38	7.84
Current Pet Ownership	Yes	283	58.35		Dog Bite	142	29.28
	No	189	38.97		Fear of Dogs	63	12.99
	Missing	13	2.68		None	227	46.80
					Missing	15	3.09

*n* = 485.

**Table 4:** Robust OLS regression with the regression coefficient and standard deviation for public access perceptions compared by independent variables (*n* = 485).

Attribute		B	*p* Value
Age	19–30	−0.103 (0.135)	0.446
	31–59	−0.132 (0.082)	0.109
	60+	Ref.	Ref.
Gender	Man	Ref.	Ref.
	Woman	0.216 (0.074)	0.004 ***
	Other Identity	0.257 (0.224)	0.251
Annual Income Level	$0 to $19,999	Ref.	Ref.
	$20,000 to $39,999	−0.051 (0.134)	0.706
	$40,000 to $59,999	−0.026 (0.1132)	0.846
	$60,000 to $79,999	−0.218 (0.131)	0.096
	$80,000 to $99,999	−0.118 (0.147)	0.422
	$100,000 to $199,999	−0.131 (0.160)	0.411
	$120,000 to $139,999	−0.450 (0.186)	0.016 **
	$140,000 or more	−0.227 (0.175)	0.196
Highest Education Level	Grade School (Elementary or High School)	Ref.	Ref.
	University Degree	0.166 (0.100)	0.098
	College or Technical Training	−0.055 (0.106)	0.603
Race/Ethnicity	Indigenous to North America/Turtle Island	0.381 (0.179)	0.034 **
	White or European	Ref.	Ref.
	Asian	−0.215 (0.157)	0.171
	Multiracial	0.163 (0.144)	0.258
	Other	−1.030 (0.144)	0.004 ***
Personal Service Dog Ownership	No	Ref.	Ref.
	Yes	−0.236 (0.223)	0.302
Personal Health Conditions	PTSD	0.050 (0.134)	0.709
	SUD	−0.197 (0.251)	0.433
	PML	0.062 (0.110)	0.570
	TBI	−0.264 (0.174)	0.131
	None Listed	Ref.	Ref.
Negative Dog Experiences	Dog Allergies	−0.081 (0.135)	0.548
	Dog Bite	−0.006 (0.083)	0.948
	Fear of Dogs	−0.209 (0.119)	0.079
	None	Ref.	Ref.
Grew Up with Pets	Yes	−0.131 (0.098)	0.184
	No	Ref.	Ref.
Current Pet Ownership	Yes	0.146 (0.073)	0.048 **
	No	Ref.	Ref.
Constant		4.33 (0.145)	0.000

Note. ** *p* < 0.05; *** *p* < 0.01; standard deviation in parenthesis; public access as a continuous variable between 1 and 5; Ran with robust estimator of variance.

**Table 5:** Predicted marginal effects of select variables on public access support (scale of 1–5).

Attribute		Margin (Predicted Response Given the Selected Attribute)	Confidence Interval (95%)
Gender	Man	4.10	4.00–4.21
	Woman	4.32 ***	4.22–4.42
	Other Identity	4.45	3.92–4.77
Race/Ethnicity	Indigenous to North America/Turtle Island	4.61 **	4.27–4.95
	White or European	4.24	4.16–4.31
	Asian	4.02	3.72–4.30
	Multiracial	4.40	4.13–4.66
	Other	3.19 ***	2.50–3.88
Current Pet Ownership	Yes	4.28 **	4.20–4.36
	No	4.13	4.01–4.25

Note. ** *p* < 0.05 and *** *p* < 0.01 in the OLS regression.

## Data Availability

The data presented in this study is currently not available. For more information, please contact the corresponding author.
